# Transurethral balloon dilatation of the Prostate and Transurethral Plasmakinetic resection of the Prostate in the treatment of Prostatic Hyperplasia

**DOI:** 10.12669/pjms.343.14516

**Published:** 2018

**Authors:** Yanhua Chang, Jingyi Chang, Hui Wang

**Affiliations:** 1 Yanhua Chang, Urology Surgery Department, Binzhou People’s Hospital, Shandong, 256610, China; 2 Jingyi Chang, First School of Clinical Medicine, Anhui Medical University, Anhui, 230032, China.; 3 Hui Wang, Urology Surgery Department, Binzhou People’s Hospital, Shandong, 256610, China

**Keywords:** Benign prostatic hyperplasia, Transurethral balloon dilatation of the prostate, Transurethral plasmakinetic resection of the prostate

## Abstract

**Background & Objective:**

With the aggravation of global aging, benign prostate hyperplasia tends to have a higher incidence and has been the most common disease in urinary surgery. It is usually treated by surgery. Our objective was to select an effective treatment scheme, the clinical efficacy and relevant indicators of transurethral balloon dilatation of the prostate (TUDP) and transurethral plasmakinetic resection of the prostate (PKRP) in the treatment of benign prostate hyperplasia were emphatically compared.

**Methods:**

Ninety-eight patients with benign prostate hyperplasia who were admitted to the hospital of between May 2014 and July 2016 were selected and divided into a TUDP group (n=49) and PKRP (n=49) using random number table. The intraoperative blood loss, duration of surgery, international prostate symptom score (IPSS), quality of life (QOL), post-void residual urine (PVR) and complications of the two groups were observed.

**Results:**

The results demonstrated that the postoperative blood loss and duration of surgery of the patients in the PKRP group were significantly higher than those of the TUDP group (P<0.05); the IPSS, QOL and PVR of the patients in the two groups after surgery were much lower than those before surgery (P<0.05); the IPSS, QOL and PVR of the patients in the PKRP group were significantly lower than those in the TUDP group after surgery (P<0.05). The incidence of postoperative complications of the PKRP group was 38.8%, which was apparently higher than 14.3% in the TUDP group (P<0.05).

**Conclusion:**

PKRP has better efficacy than TUDP in treating benign prostatic hyperplasia, but QOL was poor and there are many complications. Proper surgical procedure should be selected according to the specific disease condition of patients.

## INTRODUCTION

Benign prostatic hyperplasia is a common disease in urology surgery that can affect the quality of life of males. It manifests as lower urinary tract symptoms such as frequent micturition, urgent urination and dysuria;^1^ due to the non-typical early symptoms, prostatic hyperplasia usually has evolved to the end stage when it is diagnosed. Surgery is the main treatment method for those patients.^2^ Currently surgeries such as transurethral plasmakinetic resection of the prostate (PKRP), transurethral resection of the prostate and transurethral laser vaporization can significantly relieve lower urinary tract obstruction, which are quite effective.^3^ But the application of the above surgeries is limited due to large damages, slow postoperative recovery, large intraoperative blood loss and high incidence of complications in perioperative period.^4^ Transurethral balloon dilatation of the prostate (TUDP) as a new surgery is seldom applied in clinics, and its efficacy is still controversial.

This study observed and compared the relevant indexes of TUDP and PKRP before and after surgery and discussed the difference between the efficacy of TUDP and PKRP and the application scope and promotion values of TUDP and PKRP, aiming to guide patients and doctors to select personalized treatment scheme.

## METHODS

Ninety-eight patients with benign prostatic hyperplasia who were admitted to the hospital between May 2014 and July 2015 were selected and divided into a TUDP group (n=49) and PKRP (n=49) using random number table. In the TUDP group, they aged from 55 to 74 years (average 62.5±1.2 years) and have suffered from benign prostatic hyperplasia for 0.6~10 years (average 3.1±1.4 years); as to Rous scale, there were 8 cases of scale IV, 11 cases of scale III, 25 cases of scale II, and 5 cases of scale I. In the PKRP group, they aged from 51~76 years (average 63.6±2.1 years) and have suffered from benign prostatic hyperplasia for 0.7~10 years (average 2.9±1.7 years); as to Rous scale, there were 9 cases of scale IV, 10 cases of scale III, 24 cases of scale II, and 6 cases of scale I. There was no significant difference in the clinical baseline data between the two groups (P>0.05); hence the results were comparable. This study has been approved by the ethics committee of our hospital, and all the patients signed informed consent.

### Inclusive and exclusive criteria

Patients who were confirmed as benign prostatic hyperplasia by serum prostate-specific antigen (PSA) and B ultrasound and had repeated acute urinary retention (times of acute urinary retention ≥2), repeated hematuresis which were difficult to be controlled by hemostasia drugs, repeated urinary tract infection, secondary upper urinary tract obstruction accompanied by renal function damage and international prostate symptom score (IPSS) not lower than 8 points were included.

Patients who were extremely weak because of systemic diseases such as serious cardiopulmonary and hepatorenal dysfunction and coagulation dysfunction, had severe urinary system infection, urethrostenosis, cystolith and extremely reduced bladder volume induced by severe bladder contracture, or had neurogenic bladder diseases were excluded.

### Surgical method: Operation of TUDP

The patient was given continuous epidural anesthesia. Taking a lithotomy position, the patient’s bladder was injected with 300~500 mL of 0.9% sodium chloride solution after anesthesia. Then the urethra was expanded using F24 urethral calibrator. A catheter with metal inner core was smeared with liquid paraffin oil and inserted to the bladder. The capsula externa was injected with 25 mL of sodium chloride solution. The catheter was pulled outward for 1.5 cm after the liquid was discharged. Then the liquid in the internal capsule was discharged to reduce the pressure of the external capsule to 0.1 mPa. The tee catheter and drainage pack were connected after the metal inner core was removed. Then the pathway was washed by 0.9% sodium chloride solution. Pressure was relieved in two days; the catheter was removed after 96~120 hour of retention. Finally hemostatic drugs and antibiotics were used.

### Operation of PKRP

The patient was given continuous epidural anesthesia and continuous low pressure flush after suprapubic cystostomy. After the urethra was expanded using 24F and 27F urethral sounds, a resectoscope was inserted. Normal saline was used for washing. 22F or 24F three cavity catheter was retained after surgery. 16F nephrostomy tube was indwelled for drainage. Finally hemostatics and antibiotics were used.

### Observational indicators

The blood loss, duration of surgery and complications of the two groups were observed. Moreover the IPSS score, QOL score and PVR of the two groups were recorded before and after surgery.

### Statistical analysis

Data were analyzed using SPSS ver. 20.0. Measurement data were expressed as mean±standard deviation (SD) and processed by t-test. Enumeration data were expressed as n (%) and processed by Chi-square test. Difference was considered as statistically significant if P<0.05.

## RESULTS

### Comparison of surgical condition between the two groups

The blood loss and duration of surgery of the PKRP group was remarkably higher than those of the TUDP group (P<0.05; Table-I).

**Table-I:** Comparison of duration of surgery and blood loss (mean±SD)

Group	Duration of surgery (min)	Blood loss (mL)
TUDP group (n=53)	18.39±4.61	19.44±3.57
PKRP group (n=45)	45.40±8.23	72.27±11.09
t	16.732	28.159
P	0.000	0.000

### Comparison of various indicators between the two groups before and after surgery

The IPSS score, QOL score and PVR of the two groups after surgery were significantly lower than those before surgery (P<0.05); the IPSS score, QOL score and PVR of the PKRP group were much lower than those of the TUDP group (P<0.05; Table-II).

**Table-II:** Comparison of IPSS score, QOL score and PVR before and after surgery.

Group	IPSS (point)		QOL (point)		PVR (mL)	

	Before	After	Before	After	Before	After
TUDP group	25.7±3.8	12.2±4.2*	5.7±1.1	2.4±0.7*	97.2±16.4	28.8±10.5*
PKRP group	26.2±3.5	5.8±2.3*#	5.6±1.0	1.1±0.6*#	96.5±15.7	16.7±7.0*#

* Note: indicated P<0.05 compared to before treatment;

# indicated P<0.05 compared to the TUDP group.

### Comparison of complications between the two groups

The incidence of complications of the TUDP group was 14.3%; one patient had angina and arrhythmia during surgery but relieved after 10 minutes. During the three-month follow up after surgery, one patient had retrograde ejaculation (RE) and six patients had erectile dysfunction (ED) or aggravated ED. The incidence of complications of the PKRP group was 38.8%; four patients had malignant hypertension in perioperative period, and they were given obstruction relief and blood pressure lowering treatment; three patients had symptoms such as palpitation, short of breath, dyspnea and congestive heart failure and were sent to intensive care unit (ICU) for symptomatic treatment at the end of surgery; one patient had pulmonary edema and were given symptomatic treatment after stopping surgery; one patient felt cold because of excessive blood loss and had a low blood pressure, 80/50 mmHg, and therefore were given transfusion of 2U of blood. Moreover there were 3 cases of RE and 9 cases of ED or aggravated ED in three months after surgery. The difference in the incidence of complications had statistical significance (X^2^=5.761, P<0.05).

## DISCUSSION

Benign prostatic hyperplasia is the main reason for obstruction of urethral outlet and lower urinary tract symptoms of elderly males, and its incidence increases with age.^5^,^6^ It is usually treated by surgery. PKRP is the gold standard surgery for benign prostatic hyperplasia, but it depends highly on the comprehensive conditions of patients.^7^ Moreover it has disadvantages such as high incidence of perioperative bleeding, postoperative urethrostenosis and intraoperative transurethral resection syndrome.^8^ Therefore it has certain limitations in treating benign prostatic hyperplasia.^9^ The application of TURP provides patients with prostatic hyperplasia a new choice. Lukkarinen et al. reported that TUDP in combination with finasteride had favorable efficacy in treating prostatic hyperplasia.^10^

In this study, the postoperative bleeding amount and duration of surgery of the TUDP group were significantly lower than those of the PKRP group (P<0.05), indicating that TUDP had higher safety, shorter operation time and less blood loss. However, the improvement of IPSS score, QOL score and PVR of the PKRP was superior to that of the TUDP group (P<0.05), indicating that the overall effect of PKRP was better than that of TURP. This study analyzed the risk factors for perioperative cardiopulmonary complications and considered that the risk factors affecting cardiopulmonary complications included excessive blood loss, excessively large bladder perfusion pressure, excessively low perfusion water temperature and excessively long duration of surgical anesthesia.^11^ RE was considered because of the reduced urethral resistance after verumontanum under the effect of PKRP or the change of ejaculation direction because of verumontanum injury or dissection induced by electric resection. TUDP would not damage verumontanum; hence the incidence of RE was low. The occurrence of ED might be because of sexual nerve injury induced by cutting of prostatic capsule during TUDP.^12^

## CONCLUSION

PKRP is the preferred surgery for treating benign prostatic hyperplasia, but TUDP which is safe and effective can be regarded as the supplementary therapy for treating prostatic hyperplasia.

### Authors’ Contribution

**YHC:** Study design, data collection and analysis.

**YHC, JIC & HW:** Manuscript preparation, drafting and revising.

**YHC:** Review and final approval of manuscript.
